# Different influences on lexical priming for integrative, thematic, and taxonomic relations

**DOI:** 10.3389/fnhum.2012.00205

**Published:** 2012-07-11

**Authors:** Lara L. Jones, Sabrina Golonka

**Affiliations:** ^1^Department of Psychology, Wayne State University, DetroitMI, USA; ^2^Department of Psychology, Leeds Metropolitan UniversityLeeds, UK

**Keywords:** semantic priming, taxonomic, thematic, integrative, relational representation

## Abstract

Word pairs may be integrative (i.e., combination of two concepts into one meaningful entity; e.g., *fruit—cake*), thematically related (i.e., connected in time and place; e.g., *party—cake*), and/or taxonomically related (i.e., shared features and category co-members; e.g., *muffin—cake*). Using participant ratings and computational measures, we demonstrated distinct patterns across measures of similarity and co-occurrence, and familiarity for each relational construct in two different item sets. In a standard lexical decision task (LDT) with various delays between prime and target presentation (SOAs), target RTs and priming magnitudes were consistent across the three relations for both item sets. However, across the SOAs, there were distinct patterns among the three relations on some of the underlying measures influencing target word recognition (LSA, Google, and BEAGLE). These distinct patterns suggest different mechanisms of lexical priming and further demonstrate that integrative relations are distinct from thematic and taxonomic relations.

Lexical priming refers to faster word recognition latencies following the prior or simultaneous presentation of a meaningfully related prime word. For example, *night* would be recognized more quickly as a real word in the English language following *day, moon, dark, evening, summer*, or the indirectly related *sun*. Semantic richness refers to the variability in the information associated with a word's meaning that can facilitate lexical priming of the target following a related prime (Yap et al., 2011). There are several facets of semantic richness that include characteristics of each individual concept within a prime-target pair (i.e., item measures; e.g., frequency, length, imageability, number of senses, number of associates) as well as pair measures reflecting the relation between the pair (e.g., similarity, co-occurrence, word pair frequency). Our purpose of the current research was to demonstrate a distinction across integrative, thematic, and taxonomic relations on these pair measures. Related to this first goal, we also investigated which of these measures were related to target word recognition latencies in a lexical decision task (LDT) within each of the three relations.

## Relational taxonomies and definitions

The first step in investigating the role of relation types in lexical priming is to define, exemplify, and further establish the underlying item dimensions for each relation type. Recent relational taxonomies (Wu and Barsalou, 2009; Santos et al., 2011) include all three types of relations we will focus on in this paper—integrative, thematic, and taxonomic. Integrative relations are inferred during the process of combining two concepts into a plausible subclass of the second concept (Estes and Jones, 2006, 2009; Jones et al., 2008; *wool socks* are socks made of wool; *summer holiday* is a holiday occurring during the summer months). Integrative relations are included among the “forward phrasal associates” prime-target pairs in the Semantic Priming Project (SPP) (Hutchison et al., 2012), which is a readily available large scale study that includes various item and participant factors in addition to lexical decision and naming latencies (for review see Balota et al., 2012). They are denoted in Santos and colleagues taxonomy as “compound continuation forward.” Within what McRae and colleagues (2012) describe as the “entity” relation type, integrative relations include the internal component (e.g., *cherry pit*) and external component (e.g., *tricycle pedals*) subtype relations. Notably, earlier relational taxonomies further subdivided such integrative relations into a small and finite number of general relations (e.g., *have, for, in*; Levi, 1978), though others criticized these general relations as being overly vague (Downing, 1977; Estes and Jones, 2006).

Integrative relations have been studied more extensively in conceptual combination studies using relational priming (e.g., Gagné, 2002; Gagné and Shoben, 2002; Estes, 2003b; Gagné and Spalding, 2004, 2009; Estes and Jones, 2006; Spalding and Gagné, 2011) and memory (Jones et al., 2008; Badham et al., 2012) paradigms. Our focus within this paper is on lexical priming, in which the ability to combine the modifier or prime concept with the head noun or target concept into a plausible entity facilitates word recognition of the target word (Estes and Jones, 2009; Badham et al., 2012). As in the prior conceptual combination studies, the activation of a relation between the two concepts also underlies integrative priming.

Thematic relations refer to the link between concepts that occur together in time and space. Thematically related concepts play complementary roles in a given action or event (e.g., *needle—thread; coffee—juice*; Lin and Murphy, 2001; for review see Estes et al., 2011). The “script” relation in the SPP (Hutchison et al., 2012) includes pairs related to a common event (e.g., *rooster—farm*). They are classified by Santos et al. (2011) as an “aspect of an object or situation” and are often denoted as “event” or “situation” or “script” relations (Moss et al., 1995; Chwilla and Kolk, 2005; Hare et al., 2009; Hutchison et al., 2012; McRae et al., 2012; Metusalem et al., 2012). In turn, these event relations include object-location (e.g., *barn—hay*), and person-location (e.g., *hospital—doctor*) relations among other subtypes (Hare et al., 2009).

Taxonomic relations refer to items associated with a category and may be further divided into superordinate (category—exemplar; e.g., *animal—dog*), coordinate (two exemplars of the same category, e.g., *dog—cat*), and subordinate (e.g., *dog—beagle*). Within this study, we limit our taxonomic items to the category co-member or coordinate relations, which are denoted in the SPP as “category” relations (e.g., *cougar—lion*; Hutchison et al., 2012).

Note that these relation types are not mutually exclusive. Indeed there is much overlap with concept pairs often representing two of the three or even all three relations (e.g., *ice-cream—cake*). Integrative and thematic relations may overlap, particularly for the locative subtype of relation. For example, the concepts *hospital* and *doctor* can be integrated to denote a subclass of doctors that work in a hospital and are thematically related in that hospitals and doctors play complementary roles in a given event or situation. However, there are many other pairs that are thematic but not so integrative (e.g., *prescription—doctor*) or that are integrative but not necessarily thematic (e.g., *animal—doctor*). Integrative and taxonomic relations may overlap depending on the similarity between the concepts and, to a lesser degree, on the extent to which the concepts belong to the same *specific* category. Highly similar items that belong to a specific (or sub-) category are less likely to be integrated than less similar ones (Wisniewski, 1997; Costello and Keane, 2000; Estes, 2003a). For example, *cake* and *pie* have the same shape and both belong to the more general “food” category as well as a more specific “dessert food” category. The high similarity between these items makes them difficult to integrate. Other, less similar, items that belong to the same subcategory (e.g., *cake* and *ice-cream*) may also be considered as thematic in that they may play complementary roles in a given scenario or event (*ice-cream* and *cake* may be served together at a party). More typically though, pairs having both a thematic and taxonomic relation will be co-members of a broader category (e.g., *cake* and *coffee*; *wine* and *cheese*; *meat* and *potatoes*; “foods” or “things that can be consumed”).

## Importance of relation type on lexical priming

Many lexical priming studies have focused on the role of word association and/or feature similarity in lexical priming (Shelton and Martin, 1992; McRae and Boisvert, 1998; Thompson-Schill et al., 1998; Estes and Jones, 2009; Jones, 2010, 2012; in preparation; for review see Lucas, 2000; Hutchison, 2003; Jones and Estes, 2012). Association strength refers to the proportion of a sample in a free association task indicating a particular concept in response to a cue. For example, nearly 82% of participants in the University of South Florida Free Association norms produced *night* for the cue *day*; Nelson et al., 1998). Associations vary in strength with those having no more than 10% of a sample producing a given target considered as only weakly associated and those with more than 20% considered as strongly associated based on Hutchison's (2003) criteria. Word association strengths influence both the magnitude and even the mere presence of lexical priming (Jones, 2010, 2012; in preparation; for review see Moss et al., 1995; Nation and Snowling, 1999; Lucas, 2000; Hutchison, 2003). Therefore, word association strength must be examined as a factor, minimized, and/or equated when examining the influence of relation types on lexical priming. McRae et al. (2012) argued that equating word association strength by eliminating the most strongly associated items from the stimuli set is not an ideal solution because these items represent the best examples of a given relation. However, we chose to include only “pure” (weakly associated) prime-target pairs in the current research in order to better focus on our other variables of interest (e.g., co-occurrence, similarity), which are often related to association strength (Jones, in preparation).

In contrast to the plethora of studies examining the role of association strength, there have been far fewer studies conducted to “distinguish among types of semantic relations” in lexical priming (McRae and Boisvert, 1998, p. 568; see also McRae et al., 2012). So then further research on relations in lexical priming would fill a long-standing gap in the lexical priming literature. Such investigation is important for several reasons. First, it has implications for the development of semantic memory, which is characterized by a conceptual shift from primarily thematic, functional, or instrumental relations in young children (age < 6) to the addition of categorical (taxonomic) relations along with thematic ones beginning around age 7 (Perraudin and Mounoud, 2009; Jones and Estes, 2012; for review see Estes et al., 2011). Moreover, at least two of these relations—taxonomic and thematic—are neuro-anatomically dissociable (Sachs et al., 2008; Mirman et al., 2011; Schwartz et al., 2011). For instance, individuals with acquired language impairments resulting from brain injury or disease often exhibit specific difficulties with some relations but not others (e.g., Schwartz et al., 2011). Likewise, these relations are also expected to exhibit distinct patterns across item measures that have been found to predict lexical priming (e.g., co-occurrence, word pair frequency, similarity). These underlying measures may differentially predict lexical priming across these three relations, which would have important implications for the semantic priming models (e.g., perceptual simulation, compound cue, expectancy generation) that could account for priming effects.

In addition to distinct patterns of underlying correlates in lexical priming, there may also be differences in the magnitude of priming across relations at various SOAs. Prior studies have found evidence of more robust priming effects for thematic than taxonomic items at short SOAs (Sachs et al., 2008; Sass et al., 2009). Using a standard LDT with a short 200 ms SOA, Sachs et al. (2008) found more robust lexical priming effects (PEs; unrelated—related) for thematically related pairs (e.g., *car—garage*; PE = 57 ms) than for taxonomically related pairs (e.g., *car—bus*; PE = 39 ms), and attributed this result to a greater “salience” for the thematically related items. However, these studies did not control for word association strength, which has been shown to produce more robust lexical priming effects particularly at shorter and longer SOAs (<300 ms and > 1500 ms; Moss et al., 1995; Jones, 2012; in preparation). So then the greater salience for the thematic pairs may have actually reflected stronger word associations for the thematic than for the taxonomic pairs.

To the extent that integrative, thematic, and taxonomic relations are conceptually distinct, they should exhibit distinctive patterns across pair measures of semantic richness (e.g., co-occurrence, similarity, word pair frequency). Indeed, Maki and Buchanan (2008) found a three-factor structure across 13 underlying variables (LSA, FAS, etc.) in terms of associative, semantic, and thematic knowledge. In turn, these different types of knowledge have been found to differentially influence lexical priming in prior studies (e.g., Chwilla and Kolk, 2005; Jones and Mewhort, 2007; Hare et al., 2009). Using two different sets of items, we examine the extent to which these three relations have distinct patterns on these pair measures of semantic richness and the extent to which these underlying measures differentially predict lexical priming. Studies 1 and 2 consisted of integrative, thematic, and taxonomic prime-target pairs taken from a large-scale study (with different targets within the three relations; e.g., *tuna sandwich, patient nurse, chalk crayon*). Studies 3 and 4 consisted of a smaller set of prime-target pairs with the target held constant among the three relations (*tomato soup, bowl soup, chili soup*). For both item sets, we minimized and equated association strength and assessed local co-occurrence or word pair frequency (Google hits), and global co-occurrence (LSA cosines).

## Overview of studies 1 and 2

In Study 1, we assess the extent to which items taken from the SPP differed on two measures of global and local co-occurrence (described further in the subsequent sections) across our three relations. In Study 2, we sought to examine whether target RTs and priming magnitudes would differ across these three relations using the LDT target RTs taken from the 200 ms and 1200 ms SOAs in SPP.

### Co-occurrence

Co-occurrence between primes and targets influence lexical priming. According to compound-cue theory (Ratcliff and McKoon, 1988; McKoon and Ratcliff, 1992), faster RTs for related primes and targets are produced by the joining of prime and target to form a compound cue which is then matched against items in long-term memory. The degree of facilitation for these target RTs is based on the extent to which the prime and target are associated in memory. Co-occurrence can be assessed at varying levels. Local co-occurrence refers to the extent to which the exact prime-target word pair (e.g., *instruction book*) appears in long-term memory, whereas global co-occurrence refers to the co-occurrence of the prime and target within a given text. In the current study, we assess local co-occurrence by the frequency of the word pair in Google and global co-occurrence using LSA cosines. In addition to influencing lexical priming, the extent and type of co-occurrence is predicted to vary among the three relations.

#### LSA cosines

Latent Semantic Analysis (LSA) is a statistical approach to language learning that is able to capture subtle semantic relationships between words even though it has no knowledge of word meaning or syntax (Landauer and Dumais, 1997). The logic of the approach is that the “psychological similarity between any two words is reflected in the way they co-occur in small sub-samples of language” (Landauer and Dumais, 1997, p. 215). LSA can be applied at a number of levels—for instance, it can be used to compare texts just as well as it can be used to compare words. In general terms, LSA represents words in terms of their occurrence in particular texts. Singular value decomposition and dimension reduction filter the word vectors so that words occurring in similar or same contexts are represented similarly (Kwantes, 2005). The correlation between vectors is given by the cosine, which is a convenient proxy for the similarity between two words. LSA has successfully modeled a number of behaviors related to cognition and language use. For example, Landauer and Dumais (1997) used LSA both to model the typical vocabulary growth rate of school children and to model semantic priming effects. LSA is also able to recognize words that have the same or similar meanings (Landauer and Dumais, 1994). This reflects the multi-dimensional use of LSA in prior studies as a measure of similarity (Howard and Kahana, 2002; Gagné et al., 2005) and as a measure of more global co-occurrence (Estes and Jones, 2009; Jones, 2010, 2012). Yet Simmons [Golonka] and Estes (2006) found that LSA cosines were only moderately related to similarity ratings of word pairs (*r* = 0.36). Moreover, in an exploratory factor analysis, Maki and Buchanan (2008) found that LSA along with BEAGLE loaded on the text-based factor rather than the similarity factor. So LSA is likely a better measure of co-occurrence than a proxy for similarity.

#### Google hits

In contrast to LSA cosines, Google hits assess the local co-occurrence or word pair frequencies of the prime-target in informal written language, taking word order into account when the pair is entered in quotes in the search box. For example, “tomato soup” has a much higher number of Google hits than “soup tomato,” whereas the LSA cosines are identical for both word orders. In conceptual combination studies (Wisniewski and Murphy, 2005; Murphy and Wisniewski, 2006) and lexical priming studies (Estes and Jones, 2009; Jones, 2010, 2012), Google hits provided a measure of word pair frequency in everyday written language that was moderately correlated with familiarity ratings (*r*s = 0.50 and 0.60, Wisniewski and Murphy, 2005). Moreover, Google hits are often a better measure of local co-occurrence than familiarity ratings, which tend to be restricted in range and more variable across samples. However, this extensive variation in the number of Google hits can be problematic in that the variability may be much greater within one relation than within another. Hence, logarithmic transformed Google hits (henceforth, logGoogle) may be used to compare across different relation types (Estes and Jones, 2009; Jones, 2012). Study 1 was conducted to investigate the differences among integrative, thematic, and taxonomic relations on these measures of co-occurrence.

## Study 1

One critical difference between the integrative and the other two relations is that by definition the two concepts in integrative relations can combine into a plausible entity that denotes a subtype of the second concept (e.g., an *herb garden, rose garden*, and *vegetable garden* each denote a specific and plausible subtype of garden). Though some thematic items can be combined into a plausible entity (e.g., *playground slide, giraffe zoo*), the combined entity does not as effectively denote a subtype of the second concept (i.e., most playgrounds have slides; most zoos have giraffes). Thus, word pair frequencies (logGoogle hits) should be higher for the integrative pairs than for the thematic and taxonomic pairs. In contrast, both thematic and taxonomic pairs tend to have greater global or textual co-occurrence than the integrative items, due to the complementary roles the concepts share in a given event for the thematic items and the inclusion within the same category and high semantic similarity for the taxonomic items. Hence, global co-occurrence (LSA cosines) should be greater for the thematic and taxonomic pairs than for the integrative pairs.

### Materials

The SPP, (Hutchison et al., 2012) consists of 1661 targets selected from the Nelson et al. (1998) norms with the primary associate and a randomly selected other associate paired with each target. Primes and targets were randomly re-paired in the SPP to create unrelated items within each association group. The SPP includes extensive norms taken from the English Lexicon Project (ELP; Balota et al., 2007; http://elexicon.wustl.edu/) as well as target RTs and priming magnitudes from a LDT with a 200 ms SOA and a 1200 ms SOA. To investigate lexical priming across integrative, thematic, and taxonomic relations for only weakly associated items, we selected items having the following relations from the “Other Associates” tab in SPP: forward phrasal associates, script, and category. Next we eliminated all pairs having forward association strengths (FAS) greater than 0.10 so that only weakly associated items would be included. Results of a One-Way ANOVA confirmed equivalent and weak (all *Ms* < 0.05) FAS, *F* < 1, *p* = 0.63, and backward association strengths, *F* < 1, *p* = 0.83, across the three relations. Then we limited our items to only noun–noun prime-target pairs and removed any items having proper names for the prime or target (e.g., *hawaii hula, christmas santa*) and morphemic repetition between prime and target (e.g., *bank banker*). The final set of items used in Studies 1 and 2 consisted of 89 integrative items, 78 thematic items, and 85 taxonomic items as shown in Appendix A.

### Results and discussion

We compared the word pair frequencies (logGoogle hits) and the global/textual co-occurrence (LSA cosines) among the three relations using a One-Way ANOVA with Tukey HSD *post-hoc* tests. Results indicated reliable and robust differences among the three relations for both word pair frequencies (logGoogle hits), *F*_(2, 249)_ = 64.63, *p* < 0.001, and global co-occurrence (LSA cosines), *F*_(2, 249)_ = 13.23, *p* < 0.001. As shown in Table 1, logGoogle was highest for the integrative items, *p* < 0.001, followed by the taxonomic items, which were in turn higher than the thematic items, *p* < 0.01. In contrast, the integrative pairs had reliably lower LSA cosines than the thematic and taxonomic pairs (*p*s < 0.01), which did not differ (*p* = 0.29). In sum, these results demonstrate distinct patterns of co-occurrence for the integrative items (namely, higher word pair frequencies but lower global/textual co-occurrence) in comparison to the thematic and taxonomic relations.

**Table 1:** **Study 1, Means, Standard Deviations, Minimums, and Maximums of measures and ELP control variables**.

	**Integrative**				**Thematic**				**Taxonomic**			
	**M**	**SD**	**Min**	**Max**	**M**	**SD**	**Min**	**Max**	**M**	**SD**	**Min**	**Max**
logGoogle	6.59	0.93	3.92	9.43	5.06	0.88	2.76	7.45	5.53	0.87	2.73	8.11
LSA	0.23	0.16	0.00	0.83	0.33	0.23	-0.04	0.83	0.38	0.22	-0.01	0.92

Notes: Prime and target frequencies and baseline RTs taken from the English Lexicon Project.

## Study 2

The purpose of Study 2 was to determine whether the response times and priming effects would differ among the three relations. Recall that Sachs et al. (2008) found more robust priming for associated thematic pairs (*car—garage*) than for their associated taxonomic pairs (*car—bus*) in a standard LDT with a 200 ms SOA. Here we investigate whether such a difference would occur for our weakly associated thematic, taxonomic, and integrative items by comparing the RTs and priming effects (PEs) found in the 200 and 1200 ms SOAs of the SPP.

### Materials

The same SPP materials from Study 1 were used. Differences in prime and target lengths, frequencies, and baseline RTs (RTs for the word presented in isolation) can influence priming effects (Hutchison et al., 2008). So we compared the mean lengths, frequencies (logarithmic HAL frequencies or logHAL), and baseline RTs (taken from the ELP) for both the primes and targets across the three relations using a One-Way ANOVA with Tukey HSD *post-hoc* tests. Neither prime lengths, *F*_(2, 249)_ = 1.10, *p* = 0.33, nor target lengths, *F*_(2, 249)_ = 1.40, *p* = 0.25, differed across the three relations. However, prime frequencies differed, *F*_(2, 249)_ = 14.98, *p* < 0.001, with reliably greater frequencies for the integrative primes (*M* = 9.21, SD = 1.59) compared to the thematic (*M* = 8.20, SD = 1.45), *p* < 0.001, and taxonomic primes (*M* = 8.06, SD = 1.48), *p* < 0.001, which did not differ. Target frequencies also differed among the three relations, *F*_(2, 249)_ = 12.57, *p* < 0.001. Integrative target frequencies (*M* = 9.78, SD = 1.65) were greater than thematic (*M* = 9.14, SD = 1.28), *p* < 0.01, which in turn were marginally greater than the taxonomic targets (*M* = 8.68, SD = 1.38), *p* = 0.10. Baseline prime RTs differed among the three relations, *F*_(2, 249)_ = 5.67, *p* < 0.01, with faster RTs for the integrative primes (*M* = 627, SD = 55) than the thematic (*M* = 652, SD = 68), *p* < 0.001, and taxonomic primes (*M* = 657, SD = 70, *p* < 0.001), which did not differ, *p* = 0.83. Baseline RTs for the integrative targets (*M* = 612, SD = 50) did not differ from the thematic targets (*M* = 620, SD = 56), *p* = 0.57, but were marginally faster than the taxonomic targets (*M* = 630, SD = 61), *p* = 0.08. Baseline RTs did not differ between the thematic and taxonomic targets, *p* = 0.57. Given these differences, we next assessed whether prime frequencies, target frequencies, baseline prime RTs, and baseline target RTs were associated with our primed target RT at each SOA. Correlations with the primed target RTs at each SOA were reliable for only the target frequencies (*r* = 0.42 and *r* = 0.33 for the 200 and 1200 ms SOAs, *p*s < 0.001) and baseline target RTs (*r* = −0.35 and *r* = −0.25 for the 200 and 1200 ms SOAs, *p*s < 0.001), so we included these two variables as covariates in our analyses below. As discussed in the Introduction, we did not predict any differences among word recognition latencies or priming effects for our weakly associated items at either SOA.

### Results and discussion

We conducted two separate 3 (Relation: integrative, thematic, taxonomic; between-items) × 2 (SOA: 200, 1200; within-items) mixed ANCOVAs on the target RTs and PEs with target frequencies and baseline (ELP) target RTs as covariates. Adjusted mean RTs and PEs for each relation are shown in Table 2. Contrary to the results of Sachs et al. (2008), we found equivalent target RTs, *F*_(2, 245)_ = 1.82, *p* = 0.17, and priming effects, *F* < 1, *p* = 0.82, across the three relations. The lack of difference among the relations was consistent across both SOAs, as evident by the lack of an interaction for the RTs, *F* < 1, *p* = 0.92, and PEs, *F*_(2, 247)_ = 1.28, *p* = 0.28, nor was there an effect of SOA for either RTs, *F* < 1, *p* = 0.78, or PEs, *F*_(2, 247)_ = 1.03, *p* = 0.31. Not surprisingly, the target frequencies and baseline target RTs had a reliable effect on RTs (*p*s < 0.001), but did not impact PEs (*p*s > 0.45). No other covariates or interactions were reliable.

**Table 2:** **Study 1, Adjusted Means and (SEs) of Target RTs (ms) and Priming Effects (ms)**.

**Relation**	**200 ms SOA**		**1200 ms SOA**	
	**RT**	**Priming effect**	**RT**	**Priming effect**
Integrative	670 (6)	27^***^	699 (7)	6
Thematic	660 (7)	23^**^	684 (7)	12
Taxonomic	664 (6)	18^*^	687 (7)	24^**^

Notes: Priming Effect = Unrelated RT − Related RT.

* p ≤ 0.05;

** p ≤ 0.01;

*** p ≤ 0.001.

One-sample *t*-tests revealed reliable PEs (>0) for all relations at the 200 ms SOA (*p*s = 0.01). However, at the 1200 ms SOA, only the taxonomic items had reliable priming effects (*p* = 0.01), whereas the thematic and integrative items did not (*p* = 0.15 and *p* = 0.54, respectively). The effects for the taxonomic items are consistent with prior studies (e.g., McRae and Boisvert, 1998; Estes and Jones, 2009; for reviews see Neely, 1991; Jones and Estes, 2012) showing the rapid emergence of taxonomic priming and either the maintenance or an increase of priming magnitudes with increasing SOAs up to 1500 ms. Unfortunately, far fewer studies have investigated the maintenance of PEs for integrative and thematic items in a standard LDT with long SOAs. Estes and Jones (2009) found reliable PEs for integrative items at long SOAs of 1500, 2000, and 2500, and Jones et al. (2011) found larger PEs for integrative, thematic, and taxonomic relations at a 2000 ms SOA than at a short 100 ms SOA. However, in both of those studies, priming effects were based on the difference in target RTs following related versus non-linguistic and repetitive neutral primes (^********^). Such neutral primes tend to artificially inflate the RTs for the control condition at long SOAs, which in turn yield inflated priming effects (e.g., De Groot et al., 1982; Jonides and Mack, 1984; Jones, 2012).

These results fail to replicate the finding by Sachs et al. (2008) of different priming effects for thematic versus taxonomic items at a 200 ms SOA. Although there were no reliable differences in RTs or PEs among the relations at the 1200 ms SOA, only the taxonomic items had a reliable priming effect. The lack of priming at this longer 1200 ms SOA for the integrative and thematic items seems to preclude expectancy generation as an underlying mechanism. Indeed, the results of Jones (in preparation) suggest that strong FAS is required for integrative priming to occur for longer SOAs >1500 ms. Likewise, thematic priming for strongly associated versus weakly associated pairs may show a similar pattern with reliable priming for only the strongly associated pairs at long SOAs >1500 ms. In contrast, taxonomic priming is often attributed to semantic matching (Neely, 1991) or post-lexical integration (De Groot, 1984, 1985) which entails a search for a plausible relation between prime and target. Categorical relations would be particularly strong for our subject population of young adults attending a university (for review see Estes et al., 2011), and consequently may be better maintained in working memory over long SOAs than the integrative and thematic relations.

Finally, the inclusion of different targets across the three relations in this study and in Sachs et al. (2008) is less than ideal despite the equating or controlling of the confounding variables of target frequencies and baseline target RTs. Hence, in Studies 3 and 4, we develop a set of items so that each target (e.g., *book*) is paired with an integrative (e.g., *instruction*), thematic (e.g., *editor*), and taxonomic (e.g., *article*) prime.

## Overview of studies 3 and 4

The primary purpose of Studies 3 and 4 was to replicate and extend the results found in Studies 1 and 2 using a more controlled set of items having the same target across each relation. We begin with an item analysis to further demonstrate distinct patterns on the co-occurrence measures of LSA and logGoogle across the three relations (Study 3). As mentioned in the Introduction, we extend this item analysis to also include BEAGLE cosines, feature similarity ratings, familiarity ratings. We also include our relation defining measures of relational integration, thematic relatedness relations, and category co-membership in order to verify our classification into relational categories. Next we investigate the extent to which these measures differentially predict lexical priming across the three relations using a standard LDT with 100, 500, and 800 ms SOAs (Study 4).

## Study 3

As in Study 1, we minimized and equated association strength and assessed local co-occurrence or word pair frequency (Google hits), and global co-occurrence (LSA cosines and BEAGLE cosines). In addition to these database and computational measures, a total of 130 Wayne State University undergraduates provided ratings for categorical relatedness, thematic relatedness, integration, feature similarity, and familiarity. Each of these additional measures is described in detail below along with the relevance to semantic priming theories and the predicted differences across the three relations.

### BEAGLE cosines

The Bound Encoding of the Aggregate Language Environment (BEAGLE; Jones and Mewhort, 2007), also predicts lexical priming. Like the compound-cue model, it attributes lexical priming to the co-occurrence between prime and target. BEAGLE cosines represent a measure of the degree of shared contexts between prime and target. Pairs that are both associative and semantic (i.e., co-occurring and similar in meaning; e.g., *nurse—doctor*) are predicted to have higher BEAGLE cosines than those that are only associative (e.g., *bee—honey*) or only semantic (e.g., *deer—pony*). BEAGLE incorporates both “co-occurrence information” (i.e., information about the word's context) and “transition information” (i.e., information about a word relative to other words in a context such as the intervening words; (Jones and Mewhort, 2007, p. 5). So whereas LSA captures both similarity and textual or global co-occurrence, BEAGLE goes a step further by additionally representing transition information. Thus, given the multi-dimensional aspect of BEAGLE, cosines may be consistent across our three relations.

### Feature similarity

The features that we attend to in objects and concepts are likely to be those that help us do things like select appropriate actions and solve problems. The relation (integrative, taxonomic, thematic) between two concepts is partially determined by the distribution of common features among the items (i.e., feature similarity). Taxonomic categories are based on common features among category members (e.g., Rosch, 1975; Markman and Wisniewski, 1997). It is inherent that taxonomic category members have common properties (high feature similarity)—if they did not then taxonomic category membership could not guide particular types of inference and action in the face of incomplete information. Feature similarity can also influence the occurrence and extent of lexical priming, particularly at shorter SOAs. For instance, McRae and Boisvert (1998) found that reliable lexical priming occurred for their highly similar pairs (e.g., *goose—turkey*) at a 250 ms SOA but not for the less similar pairs (e.g., *robin—turkey*). Thematically related items are often based on the ability of the items to play complementary roles in the same scenario (Lin and Murphy, 2001), which is facilitated (but not necessitated) by items having non-overlapping features (e.g., *cake*—*ice*-*cream* is more thematically related than *cake*—*pie*). However, many thematically related pairs (e.g., *prescription—doctor*) are based primarily on their complementary roles in the same event and are not dependent on the extent of overlapping features between items. Item pairs that share very few common features are possible candidates for integrative relations. Integrating two concepts into a single, modified concept requires very low overlap in features between items (Estes, 2003a).

As in Estes and Jones (2009), participants (*N* = 25) rated the feature similarity of each word pair on a scale from 1 (not at all similar) to 7 (very similar). Feature similarity was emphasized in the instructions and differentiated via examples from association and co-occurrence. Instructions for this and all subsequent rating tasks are included in Appendix B. Based on the prior research described above, we predicted that feature similarity would be highest for the taxonomic pairs and lowest for the integrative pairs with the thematic pairs having a feature similarity intermediate between these two other relations.

### Familiarity ratings

As an additional measure of local co-occurrence or word pair frequency, participants (*N* = 21) rated the familiarity for each pair on a scale from 1 (unfamiliar) to 7 (very familiar). We also assessed the familiarity of our prime-target pairs. As previously mentioned, familiarity is moderately correlated with Google hits. In addition to highly frequent word pairs, familiarity is also likely to be high for words that seem to go together in a given event (e.g., *party—cake*). Hence, we predict higher familiarity ratings for the integrative and thematic items than for the taxonomic pairs.

### Relation verification ratings

In order to select a final set of the most representative items possible for each relation and to verify our designation of each word pair as taxonomic, thematic, or integrative, we collected category co-membership, thematic relatedness, and integrative ratings, respectively. In making our selection of items to include in the final set, we adopted the criteria that the rating measure should be equal to or greater than the midpoint of 4.00 (on a scale of 1–7) for the respective measure representing that relation (e.g., all thematic items should have a thematic relatedness rating of 4 or greater). Additionally, each of the three measures should be reliably higher for the items in the represented relation than for the items in the other two relations (e.g., thematic relatedness ratings should be reliably higher for the thematic than for the taxonomic or integrative items). For each of the following three rating tasks, the 60 targets were presented with each of their prime-types and the presentation order of all 180 items was randomized across participants.

#### Categorical co-membership ratings

Because category membership is based on more than just feature similarity (e.g., Spalding and Ross, 2000), we needed to directly assess the extent to which each prime and target belonged to the same specific taxonomic category. Participants (*N* = 28) rated each pair from 1 (not at all category co-members) to 7 (definitely co-members of the same specific category). Instructions distinguished taxonomic relatedness over thematic relatedness and relational integration by emphasizing co-membership in a specific category (see Appendix B).

#### Thematic relatedness ratings

Participants (*N* = 27) rated the extent to which each pair of concepts was linked together in a common scenario, event, or function on a scale from 1 (not thematically connected) to 7 (highly thematically connected). Instructions emphasized that thematically related concepts were often not featurally similar (see Appendix B).

#### Relational integration ratings

To better distinguish integrative relations from thematic relations we used the sentential integrative rating task from Estes and Jones (2009), which was found in that study to be highly correlated with integrative ratings for the isolated word pair (*r* = 0.80). Participants (*N* = 29) rated the extent to which the word pair made sense as an object within a sentential context from 1 (not at all sensible) to 7 (completely sensible). The same sentence frame was used for each target across the three relations with the word pair shown in ALL CAPS as the object of each sentence (e.g., “Irene ordered the CHILI SOUP”—taxonomic; “Irene ordered the BOWL SOUP”—thematic; “Irene ordered the TOMATO SOUP”—integrative). Note that in this integrative rating task, the integrative pairs (e.g., *tomato soup*) should have much higher ratings than the thematic pairs, which are not as readily integrative (e.g., *bowl soup* does not easily denote a subtype of soup, as soup is typically served in a bowl).

### Materials

Based on the results from the three relational verification rating tasks, we narrowed down the prior set of 180 items (60 per relation) to a final set of 132 items (44 per relation) in order to better minimize the degree to which items could represent more than one relation. This final set of items is shown in Appendix C.

### Results and discussion

The means, SDs, minimums, and maximums on each of these measures (5 rating tasks and 3 computational measures) are shown for each relation in Table 3. Separate One-Way ANOVAs and LSD *post-hoc* tests (see Table 4) with the relation representative measures of integrative ratings, thematic relatedness, and category co-membership confirm that: (1) the integrative items had higher integrative ratings than did the taxonomic and thematic items, (2) the thematic items had higher thematic relatedness ratings than the integrative and taxonomic items, and (3) the taxonomic items had higher category co-membership ratings than the other two relations. Moreover, as shown in Table 4, separate One-Way ANOVAs on the remaining measures revealed reliable differences among the three relations for feature similarity ratings, LSA, and familiarity ratings, but only marginally for logGoogle, and not for BEAGLE. Unsurprisingly, feature similarity ratings were higher for the taxonomic items than for the thematic items, which in turn were higher than those for the integrative items. As in Study 1, LSA cosines were lower for the integrative items in comparison to the taxonomic and thematic items, which were equivalent. In addition to reflecting similarity of the taxonomic items, the high LSA cosines may have simply reflected the fact that members of a given category often co-occur within the same text. Familiarity ratings were higher for the thematic than the integrative items, which in turn were higher than the taxonomic ratings. Word pair frequencies (logGoogle) hits were higher for the integrative items than the taxonomic items, but were equal to the thematic items. The lack of difference between the thematic and integrative items may reflect the ability to integrate several of the thematic pairs into a sensible entity (e.g., *lab coat, jelly jar*).

**Table 3:** **Study 3, Means, Standard Deviations, Minimums, and Maximums of Measures**.

**Measure**	**Integrative**				**Thematic**				**Taxonomic**			
	**M**	**SD**	**Min**	**Max**	**M**	**SD**	**Min**	**Max**	**M**	**SD**	**Min**	**Max**
Integrative ratings	5.50	0.82	4.00	6.69	4.79	1.14	2.97	6.76	3.81	1.04	1.97	6.28
Thematic relatedness	4.69	0.82	2.93	6.33	5.59	0.63	4.19	6.52	4.92	0.67	3.85	6.30
Category co-members	3.74	0.74	2.25	5.68	4.37	0.63	3.00	5.57	4.95	0.54	4.00	6.00
Feature similarity	3.36	0.74	2.00	5.08	3.78	0.66	2.60	5.48	5.37	0.53	4.16	6.28
Familiarity	5.63	0.79	3.67	6.76	5.97	0.45	4.90	6.81	5.14	0.74	3.24	6.24
logGoogle	5.21	0.67	2.71	6.08	5.08	0.49	3.47	5.88	4.95	0.53	3.73	6.11
BEAGLE	0.27	0.14	0.02	0.66	0.29	0.15	-0.01	0.71	0.31	0.17	0.04	0.65
LSA	0.27	0.18	0.04	0.79	0.38	0.19	0.07	0.72	0.39	0.19	0.09	0.75

**Table 4:** **Study 3, differences among relations for each measure**.

**Measure**	**ANOVA**	**Comparisons (*Post-hoc*, LSD)**
Integrative ratings	*F* = 39.02, *p* < 0.001	Integrative > Thematic > Taxonomic
Thematic relatedness	*F* = 31.20, *p* < 0.001	Thematic > (Taxonomic = Integrative)
Category co-members	*F* = 18.94, *p* < 0.001	Taxonomic > Thematic > Integrative
Feature similarity	*F* = 117.12, *p* < 0.001	Taxonomic > Thematic > Integrative
Familiarity	*F* = 16.36, *p* <0.001	Thematic > Integrative > Taxonomic
logGoogle	*F* = 2.38, *p* < 0.10	**Integrative > Taxonomic,** Integrative = Thematic, Taxonomic = Thematic
BEAGLE	*F* < 1, *p* = 0.42	Taxonomic = Thematic = Integrative
LSA	*F* = 5.75, *p* < 0.01	**(Taxonomic = Thematic) > Integrative**

Notes: Comparisons shown in **bold** font replicate results found in Study 1.

#### Predictor variable inter-correlations

The inter-correlations among these measures for all 132 items are shown in Table 5. In the next few sub-sections, we highlight some of the correlations that show further distinction across our three relations.

**Table 5:** **Study 3, Inter-correlations of ratings and computation measures**.

	**1**	**2**	**3**	**4**	**5**	**6**	**7**	**8**
Integrative ratings	−	−	−	−	−	−	−	−
Thematic relatedness	0.04	−	−	−	−	−	−	−
Category co-members	−0.25^**^	0.60^***^	−	−	−	−	−	−
Feature similarity	−0.46^***^	0.17^†^	0.74^***^	−	−	−	−	−
Familiarity	0.50^***^	0.63^***^	0.22^*^	−0.16^†^	−	−	−	−
logGoogle	0.18^*^	0.09	−0.04	−0.06	0.33^***^	−	−	−
BEAGLE	−0.21^*^	0.08	0.20^*^	0.18^*^	0.08	0.42^***^	−	−
LSA	−0.19^*^	0.30^***^	0.36^***^	0.26^**^	0.14	0.15^†^	0.49^***^	−

Notes:

† p < 0.10,

* p ≤ 0.05,

** p ≤ 0.01,

*** p ≤ 0.001.

#### Inter-correlations with integrative ratings

Despite the overlap between integrative and thematic relations in general and for some of our items (e.g., *ambulance siren, shower soap*), we found no overall relationship between integrative and thematic ratings across our item set. The integrative ratings and category co-member (i.e., taxonomic) ratings were inversely related. Likewise, the inverse relationships between feature similarity and integrative ratings across all items are consistent with the dissociation between integrative (a.k.a., “relational”) and taxonomic (a.k.a., “attributive”) pairs observed in lexical priming (Estes and Jones, 2009, Experiment 2) and conceptual combination (Wisniewski and Love, 1998; Estes, 2003b) studies. For instance, across the 45 integrative and 45 “semantic” (i.e., taxonomic) items used by Estes and Jones, there was an inverse relationship between the sentential integrative ratings and feature similarity ratings (*r* = −0.55, *p* < 0.001). These inverse correlations further underscore the difficulty (but not impossibility) of relationally integrating two highly similar items from the same category (e.g., *cow horse, lake ocean, knife spoon*). Yet, as mentioned in the Introduction, there is also overlap between taxonomic and integrative relations. Despite our best efforts to tease apart the three relations in the creation of our item set, this overlap was reflected by a few items of our taxonomic and integrative pairs (e.g., *alarm siren*, *pork bacon*, *suit pants, chocolate candy*) that had high ratings across category co-membership, feature similarity, and integration. These items likely reduced the extent to which integrative ratings were inversely correlated with category co-membership and feature similarity. As shown in Table 5, integrative ratings were positively and robustly associated with familiarity, though only weakly related to logGoogle hits. However, integrative ratings were inversely related to the more global co-occurrence measures of BEAGLE and LSA cosines.

#### Inter-correlations with thematic relatedness ratings

In contrast, thematic relatedness was positively associated with category co-membership. This positive association is consistent with Lin and Murphy (2001), who argued that thematic relations (e.g., *chalk/blackboard*) sometimes create more coherent categories than taxonomic relations (e.g., *chalk/marker*). However, as demonstrated by the merely marginal correlation between thematic relatedness and feature similarity, members of thematic categories do not cohere around shared features. Rather, members of thematic categories are united by playing complementary roles in the same scenario or event (Estes et al., 2011). The correlation between thematic relatedness and feature similarity is relatively weak because objects that have the same properties and affordances are unlikely to be able to engage in a complementary action (although for some exceptions see Wisniewski and Bassok, 1999). Thematic ratings were also robustly correlated with familiarity but not with logGoogle or BEAGLE. Hence, subjective familiarity reflects not only the ability to integrate two concepts, but also (and to a slightly greater degree) the co-occurrence of the concepts within an event. However, in contrast to the inverse correlation with the integrative ratings, LSA cosines were positively associated with thematic relatedness. Hence, the respective correlations with LSA cosines further distinguish between thematic and integrative relations.

#### Inter-correlations with category co-membership ratings

Not surprisingly, category co-membership was strongly and positively associated with feature similarity ratings. This robust correlation is consistent with models of categorization and prior studies. Family resemblance approaches to category coherence are based on the tenet that taxonomic categories cohere around common features (Rosch, 1975; Rosch and Mervis, 1975). The importance of feature similarity to category structure is reflected in the relationship between category membership and perceived similarity. Category co-members like *milk* and *lemonade* are regularly judged to be more similar to one another than category non-members like *milk* and *horse* (Murphy and Brownell, 1985; Wisniewski and Bassok, 1999; Golonka and Estes, 2009). Thus, category membership was strongly related to similarity. In contrast to the integrative and thematic ratings, category co-membership was only weakly related to familiarity. Consistent with the thematic relatedness ratings, category co-membership was not related to logGoogle hits but was reliably related to LSA cosines. In contrast to the inverse correlation with integrative ratings, and the lack of an association with the thematic ratings, BEAGLE cosines were related (albeit weakly) to category co-membership.

#### Inter-correlations among the co-occurrence measures, similarity ratings, and familiarity

Though not a primary goal of our study, we briefly highlight some of the inter-correlations that replicate interesting patterns found in prior studies. As discussed in Study 1, it is increasingly common to use LSA cosines as a proxy for similarity. However, like Simmons [Golonka] and Estes (2006), we found only a weak association between LSA cosines and feature similarity ratings. In support of our claim that LSA is a better measure of textual co-occurrence than similarity, LSA cosines were more strongly correlated with BEAGLE (*r* = 0.49) in comparison to feature similarity ratings (*r* = 0.26). This finding also corroborates the results of Maki and Buchanan's (2008) exploratory factor analysis, which found that LSA along with BEAGLE more strongly loaded on the text-based factor rather than the similarity factor. As with LSA, BEAGLE cosines were only weakly related to feature similarity ratings.

In direct contrast to LSA cosines, logGoogle hits were reliably related to integrative ratings but not to thematic relatedness or category co-membership ratings. Also, in direct contrast to the two more global co-occurrence measures (LSA and BEAGLE cosines), logGoogle was not related to feature similarity ratings. The three co-occurrence measures (logGoogle, BEAGLE, and LSA) were interrelated, though to a much lesser extent between logGoogle and LSA. The correlation between BEAGLE and LSA is consistent with that found by Jones and Mewhort (2007, Table 5; *r* = 0.37). Moreover, familiarity ratings were related to logGoogle, consistent with the findings of Wisniewski and Murphy (2005), but not to BEAGLE or LSA. The finding that BEAGLE was more related to LSA and to logGoogle (both *r*s > 0.40) than these two measures were to each other indicate that BEAGLE cosines reflect both local and global co-occurrence. Indeed, this finding supports the BEAGLE model's incorporation of both “co-occurrence information” (i.e., information about the word's context) and “transition information” (i.e., information about a word relative to other words in a context; Jones and Mewhort, 2007, p. 5). In Study 4, we predict that the various co-occurrence measures (logGoogle, LSA cosines, BEAGLE) will differentially predict lexical priming across the three relations.

## Study 4

As shown in Study 3, global measures of co-occurrence (LSA and BEAGLE) were particularly high for both the taxonomic and thematic pairs. For these items, we predict that the more global co-occurrence measures should facilitate priming effects by facilitating global integration (Chwilla and Kolk, 2005), or expectancy processing, in which an upcoming target is anticipated based on its frequent inclusion in an event (McRae and Matsuki, 2009; Metusalem et al., 2012). For instance, Chwilla and Kolk attributed lexical priming in a LDT with a short SOA for target items following two simultaneously script-related primes (e.g., *move—piano → backache*) to their global integration model and to higher LSA cosines for their script-related items than their unrelated items. A similar study (Khalkhali et al., 2012) attributed lexical priming for targets following individually presented primes depicting events that occurred prior to the target event (e.g., *marinate → grill → chew*) to the integration of the prime concepts into a situation model (i.e., a mental representation of a sequence of events). As with Chwilla and Kolk (2005), LSA cosines were also higher for the related than the unrelated triplets. Likewise, Jones and Mewhort (2007) found that BEAGLE cosines predicted priming for the semantic (mostly taxonomic) non-associative pairs (e.g., *deer—pony*) used in Chiarello et al. (1990). So then, these findings tentatively suggest that global co-occurrence (LSA and BEAGLE cosines) may predict target word recognition latencies following thematic and taxonomic primes.

For the integrative items, word pair frequencies (logGoogle hits) should predict lexical priming, particularly at short SOAs. The Embodied Conceptual Combination (ECCo) model (Lynott and Connell, 2010) posits a “quick and dirty” linguistic shortcut in which interpretation times (and by extension word recognition times) are faster for more frequently co-occurring combinations. This theory of conceptual combination interpretation is congruent with the compound-cue theory in lexical priming (Ratcliff and McKoon, 1988; McKoon and Ratcliff, 1992) which argues that prime-target compounds that are highly co-occurring in long-term memory produce faster RTs than less accessible ones. Hence, based on the ECCo and compound cue theories, logGoogle should influence target RTs, but only at the short 100 ms SOA.

### Method

#### Participants

Wayne State University undergraduates (*N* = 223) participated for partial course credit and were randomly assigned to the 100 ms SOA (*N* = 57), the 500 ms SOA (*N* = 105) or the 800 ms SOA (*N* = 61).

#### Materials

Experimental items consisted of the final set of items from Study 3 (see Appendix C). As in Study 2, prime frequencies (logHAL), length, and RTs were taken from the ELP website (Balota et al., 2007, http://elexicon.wustl.edu/) and compared across the three relations. A One-Way ANOVA found no reliable differences among the relations for prime length, *F* < 1, *p* = 0.78, or prime RTs, *F*_(2, 126)_ = 1.05, *p* = 0.35. However, prime frequencies differed among the relations, *F*_(2, 126)_ = 3.12, *p* < 0.05, with reliably greater frequencies for the integrative primes (*M* = 9.10, SD = 1.49) and marginally greater frequencies for the thematic primes (*M* = 8.92, SD = 1.30) in comparison to the taxonomic primes (*M* = 8.33, SD = 1.68).

#### Procedure

Participants responded only to the target words. On each of four experimental lists, critical trials consisted of 44 real word targets following an integrative prime (11 trials), thematic prime (11 trials), taxonomic prime (11 trials), or an unrelated prime (11 trials). An additional 44 filler trials consisted of a real word prime followed by a non-word target (e.g., *page—hife*). As in prior studies (e.g., Jones, 2012), non-word primes were selected from the ELP (Balota et al., 2007) so that they would not differ in length from the real word primes. Prime-types were counterbalanced across lists. Primes were vertically and horizontally centered in 22-point red Arial font on a black screen and targets were in white font. Participants pressed the spacebar to begin each trial. A blank screen appeared for 200 ms followed by a fixation plus sign (+) for 500 ms. Next the prime word appeared for 100 ms immediately followed by the target in the 100 ms SOA condition or by a blank screen for 400 ms in the 500 ms SOA or 700 ms in the 800 ms SOA. Targets remained on the screen until participants indicated whether the letter string was a real word by pressing the J key for “yes” or the F key for “no.” A 1000 ms inter-trial interval separated each trial, and presentation order of the 88 trials was randomized across participants. Ten practice trials preceded the 88 experimental trials.

### Results and discussion

RTs from incorrect trials (1.4% of the data) were excluded from analyses in addition to RTs greater than 1500 ms and any remaining RTs greater than 2.5 SDs above or below each participant's condition mean (an additional 5.6%). Mean response times and accuracies were analyzed using a 3 (SOA: 100, 500, 800; between-participants) ×4 (Prime-type: integrative, taxonomic, thematic, unrelated; within-participants) ANOVA across participants *F*_p_ and items *F*_i_. All factors were within items. Accuracies were at ceiling (all *M*s = 0.98) and there were no reliable main effects or interactions (*p* > 0.20).

Mean RTs and priming effects are shown in Table 6. Overall, RTs were slower for targets following the unrelated primes than for targets following the three related primes, *F*_p (3, 660)_ = 11.96, *p* < 0.001, η^2^_p_ = 0.05, and *F*_i (3, 129)_ = 10.28, *p* < 0.001, η^2^_p_ = 0.19. There was no main effect of SOA by subjects, *F*_p (2, 220)_ = 1.68, *p* = 0.19. Within the item analysis, RTs did not differ between the 500 ms and 800 ms SOAs (both *M*s = 667), but were faster than in the 100 ms SOA (*M* = 701), *F*_i (2, 86)_ = 50.69, *p* < 0.001. There was not an interaction between SOA and Relation, *F*_p (6, 660)_ < 1, *p* = 0.51, and *F*_i (6, 258)_ = 1.35, *p* = 0.24.

**Table 6:** **Study 4, Means and (SEs) of RTs (ms), Priming Effects (ms), and Predictors of Target RTs and Priming Effects**.

	**100 ms SOA**		**500 ms SOA**		**800 ms SOA**	
**Relation**	**RT**	**Priming effect**	**RT**	**Priming effect**	**RT**	**Priming effect**
Unrelated	722 (15)	–	689 (15)	–	687 (14)	–
Integrative	700 (14)	22^*^	671 (13)	18^*^	654 (13)	33^*^
Thematic	690 (14)	32^**^	671 (13)	18^*^	661 (14)	26^**^
Taxonomic	694 (16)	26^**^	655 (13)	34^**^	660 (15)	27^**^

Notes: Priming Effect = Unrelated RT − Related RT.

* p ≤ 0.05;

** p ≤ 0.01.

To determine whether there were differences in priming magnitude among the integrative, thematic, and taxonomic relations, we ran a 3 (SOA) × 3 (Relation) ANOVA with priming effects (unrelated RTs—related RTs) as the dependent measure. Priming effects did not differ among the three relations, *F*_p (2, 440)_ < 1, *p* = 0.56, and *F*_i (2, 86)_ < 1, *p* = 0.54, or among the SOAs, *F*_p(2, 220)_ < 1, *p* = 0.86, and *F*_i (2, 86)_ < 1, *p* = 0.88, nor was there a reliable interaction, *F*_p (4, 440)_ = 1.28, *p* = 0.28, and *F*_i (4, 172)_ = 1.96, *p* = 0.10. The consistent priming effects among all three relations across the SOAs replicates the pattern of results found in Study 2 and in our earlier study using neutral primes (Jones et al., 2011). Hence, in contrast to the results of Sachs and colleagues (2008), thematic priming was not more robust than taxonomic priming.

#### Partial correlations and regression analyses

Though priming effects were equivalent among the three relations within each SOA, different underlying measures were related to priming within each relation at each SOA (see Table 7). Because prime frequencies and prime latencies can influence target RTs (Hutchison et al., 2008), especially at short SOAs, we controlled for prime logHAL frequencies (which differed among the three relations—see “Materials” section) and baseline prime RTs (taken from the ELP). Given the numerous factors that influence target word recognition times (e.g., frequencies, orthographic neighborhoods, etc.), we also included the baseline target RTs (also taken from the ELP) as a control variable. For the partial correlation analyses reported below, we examined the influence of several common factors related to word recognition latencies, namely, feature similarity (e.g., McRae and Boisvert, 1998), LSA (e.g., Hare et al., 2009; Jones, 2012), BEAGLE (e.g., Jones and Mewhort, 2007; Hare et al., 2009), and Google hits (e.g., Jones, 2010, 2012). All marginal (*p* < 0.10) and reliable (*p* < 0.05) predictors found in the partial correlation analyses were then further examined in hierarchical stepwise regression analyses for the applicable SOA's target RTs with the same control variables (prime frequencies, baseline prime RTs, and baseline target RTs) entered into the first block and the marginal and reliable correlates entered into the second block. Variables that had beta coefficients with significance levels greater than 0.05 were excluded from the best fitting model.

**Table 7:** **Study 4, partial correlations—predictors of target response times (ms) by relation**.

	**100 ms SOA**	**500 ms SOA**	**800 ms SOA**
**INTEGRATIVE ITEMS**			
Feature similarity	−0.28^†^	−0.06	−0.18
logGoogle	−0.47^**^	−0.18	−0.25
BEAGLE	−0.23	−0.15	−0.18
LSA	−0.18	−0.001	−0.15
**THEMATIC ITEMS**			
Feature similarity	0.06	0.16	−0.31^*^
logGoogle	−0.02	−0.34^*^	−0.03
BEAGLE	0.16	−0.16	−0.33^*^
LSA	0.19	0.04	−0.26^†^
**TAXONOMIC ITEMS**			
Feature similarity	−0.17	−0.24	−0.16
logGoogle	−0.11	−0.36^*^	−0.11
BEAGLE	−0.08	−0.22	−0.32^*^
LSA	0.19	−0.12	0.02

Controlled for the following variables taken from the English Lexicon Project (ELP): Prime (HAL) logFrequency, Prime RTs, Target RTs. Note:

† p ≤ 0.10;

* p ≤ 0.05;

** p ≤ 0.01.

#### Integrative items

Local co-occurrence (word pair frequencies) as measured by logGoogle was reliably related to the target RTs at the 100 ms SOA. Additionally, we found a marginal correlation between feature similarity ratings and target RTs at this short SOA. No other variables approached conventional levels of significance. These correlates (logGoogle and feature similarity ratings) and the criterion measure of target RTs for the 100 ms SOA were entered into a hierarchical stepwise regression model with only the control variables entered in the first block and the predictors added to the second block. Results demonstrated that the inclusion of logGoogle (β = −0.42, *t* = 2.71, *p* = 0.01) along with baseline target RTs (β = 0.34, *t* = 2.18, *p* < 0.05) as the best fitting model, *r* = 0.63, *R*^2^ = 0.40, *F*_(2, 40)_ = 13.28, *p* < 0.001. Moreover, the addition of logGoogle was reliably more predictive of target RTs in the 100 ms SOA than only the baseline target RTs in Model 1, Δ *R*^2^ = 0.17, *F*_(1, 40)_ = 11.04, *p* < 0.01. Either compound-cue theory or the ECCo theory may explain these effects at this short 100 ms SOA. That is, the more familiar or frequently co-occurring the word pair, the easier it is to retrieve the representation of that entity from memory. The current results suggest a very rapid process of retrieval consistent with the ECCo model and compound-cue theories.

#### Thematic items

In contrast to the results for the integrative items, no predictors were reliably related to target RTs at the 100 ms SOA. Word pair frequencies (logGoogle) were related to target RTs at only the 500 ms SOA but did not approach significance at the shorter 100 ms or the longer 800 ms SOAs. Interestingly, the co-occurrence measures (LSA and BEAGLE) and feature similarity were related to word recognition target RTs at the 800 ms SOAs (albeit only marginally for LSA) but not at the 100 or 500 ms SOAs. As before, within the applicable SOAs, we included the marginal and reliable correlates along with the control variables in the hierarchical stepwise regression analyses to determine whether these co-occurrence variables explained the variance in target RTs above and beyond the control variables. Within the 500 ms SOA, the best fitting model included baseline target RTs (β = 0.35, *t* = 2.63, *p* = 0.01), baseline prime RTs (β = 0.32, *t* = 2.39, *p* < 0.05), and logGoogle (β = −0.28, *t* = 2.16, *p* < 0.05), *r* = 0.58, *R*^2^ = 0.33, *F*_(3, 39)_ = 6.50, *p* = 0.001. The addition of logGoogle to the model explained more of the variance than just the two control variables alone, Δ *R*^2^ = 0.08, *F*_(1, 39)_ = 4.69, *p* < 0.05. Within the 800 ms SOA, the best fitting model included only the baseline target RTs (β = 0.36, *t* = 2.59, *p* = 0.01) and BEAGLE cosines (β = −0.31, *t* = 2.26, *p* < 0.05), *r* = 0.54, *R*^2^ = 0.29, *F*_(2, 40)_ = 8.30, *p* = 0.001. Moreover, the inclusion of BEAGLE cosines accounted for more of the variance than baseline target RTs alone, Δ *R*^2^ = 0.09, *F*_(1, 40)_ = 5.11, *p* < 0.05.

Our results for the 800 ms SOA corroborate those of prior studies (Chwilla and Kolk, 2005; Hare et al., 2009), which also found an influence of global co-occurrence (LSA and BEAGLE) for most thematic relations. The influence of global co-occurrence measures like BEAGLE on target RTs reflects the activation of event knowledge, because words that are related to a common event co-occur (Hare et al., 2009). Notably, only word pair frequencies (logGoogle) were predictive beyond the control variable at the 500 ms SOA, which suggests an initial attempt at a more local integration between the two concepts prior to a more global integration of the two concepts within an event at the 800 ms SOA. The time course of activation of such event knowledge in our standard LDT is consistent with that found in other word recognition studies (e.g., Chwilla and Kolk, 2005). For instance, Chwilla and Kolk found faster RTs and an N400 priming effect for targets following two non-associated script related primes (e.g., *backache* following the simultaneously presented primes *move* and *piano*). These primes were presented for 400 ms and the N400 effect occurred an additional 400–500 ms following target presentation for a total duration of approximately 800 ms following prime onset. Hence, our results are consistent with the global integration model proposed by Chwilla and Kolk (2005) or formation of a situation model (Khalkhali et al., 2012), in which global co-occurrence rather than local co-occurrence facilitates the integration of prime and target. Alternatively, expectancy generation (Metusalem et al., 2012) may also account for our results via the formation (given ample time) of a small set of anticipated event-related targets prior to target presentation (e.g., *bacon, breakfast, toast* following *eggs*). Most importantly, these results are the first to demonstrate a key difference in the underlying influences of lexical priming between integrative pairs (e.g., *turkey bacon*) and thematic pairs (e.g., *eggs bacon*).

#### Taxonomic items

The pattern of results for the taxonomic items across the three SOAs was somewhat similar to that of the thematic items. Within the 500 ms SOA, only word pair frequencies (logGoogle) were reliably correlated with target RTs. Yet in contrast to the thematic items, only the BEAGLE cosines were reliably related to target RTs within the 800 ms SOA. In the regression analyses, the pattern of results was identical to that for the thematic items. Within the 500 ms SOA, the best fitting model included baseline target RTs (β = 0.55, *t* = 4.57, *p* < 0.001) and logGoogle, (β = −0.29, *t* = 2.44, *p* < 0.05), *r* = 0.67, *R*^2^ = 0.45, *F*_(2, 40)_ = 16.09, *p* < 0.001. The addition of logGoogle accounted for additional variance in target RTs, Δ *R*^2^ = 0.08, *F*_(1, 40)_ = 5.96, *p* < 0.05. Within the 800 ms SOA, the best fitting model included baseline target RTs and BEAGLE cosines, *r* = 0.43, *R*^2^ = 0.18, *F*_(2, 40)_ = 4.47, *p* < 0.05. The addition of BEAGLE cosines accounted for additional variance in target RTs, Δ *R*^2^ = 0.08, *F*_(1, 40)_ = 4.01, *p* = 0.05. Moreover, in this model the BEAGLE cosines (β = −0.30, *t* = 2.00, *p* = 0.05) were a reliable predictor, whereas the baseline target RTs were not (β = 0.22, *t* = 1.46, *p* = 0.15).

Results corroborate Jones and Mewhort's (2007) finding that BEAGLE cosines predicted priming for non-associated taxonomic items. The correlation between feature similarity and taxonomic target RTs was not reliable in any of the SOAs, though the trend was in the predicted direction. The lack of a reliable correlation may simply reflect the range restriction for feature similarity across these uniformly and highly similar taxonomic items (see minimums and maximums in Table 3).

## General discussion

We demonstrated distinct patterns of underlying factors (e.g., local and global co-occurrence) among weakly associated integrative, thematic, and taxonomic pairs for a large item set with different targets taken from the SPP (Study 1) and for a smaller more controlled item set having the same target across the relations (Study 3). Most notably, our results were the first to demonstrate a distinction between integrative pairs (e.g., *turkey bacon*) and thematic pairs (e.g., *eggs bacon*), with relatively less distinction between the thematic and taxonomic items. Integrative pairs were lower in global co-occurrence (LSA cosines; cf. Studies 1 and 3) and feature similarity (Study 3) in comparison to thematic and taxonomic pairs. We also found distinct patterns of correlations between each of the relational classification ratings (integrative, thematic, and category co-membership) and the other measures, thereby further distinguishing among these relations (see Table 5). The integrative ratings were *inversely* related to category co-membership, feature similarity, and LSA cosines, whereas thematic relatedness ratings were *directly* related to these measures (though only marginally related to feature similarity). This distinction between integrative and thematic relations is an important finding for semantic relation researchers using both behavioral and neuroscience methods. For instance, would additional areas of brain activation result for pairs that are both integrative and thematic? On a related note, could the earlier activation obtained for thematic in comparison to taxonomic pairs in earlier studies (e.g., Sachs et al., 2008; Sass et al., 2009) be partially attributed to the ability to integrate some of the thematic items? For example, weakly associated thematic items that can also be easily integrated, as is the case with many locative relations (e.g., *hospital doctor*), may exhibit distinct priming characteristics, time courses of activation, and/or underlying neural regions of activation in comparison to thematic relations that are less easily integrated (e.g., *prescription doctor*).

Despite the distinct pattern on these measures for each relation we found no differences in overall priming magnitudes among the relations in Studies 2 and 4. Recall that prior studies had previously shown such a dissociation between thematic and taxonomic relations in regards to time course and/or strength of activation (generally with earlier and/or more robust activation for thematic than taxonomic items Sachs et al., 2008; Sass et al., 2009; Mirman et al., 2011; Mirman and Graziano, in press). Though priming effects were equivalent among the three relations, the underlying measures of logGoogle and BEAGLE cosines differentially predicted the observed priming within each relation across the three SOAs in Study 4. In turn, the distinct patterns of predictors across the three relations suggest that different priming mechanisms were responsible for each relation. For the integrative items, local co-occurrence (logGoogle) predicted integrative priming at the 100 ms SOA. This finding suggests a “short-cut” in which the integrated prime-target pair may be retrieved from memory similar to that found in conceptual combination interpretation (Lynott and Connell, 2010) or to the compound-cue model in semantic priming (McKoon and Ratcliff, 1992). Consistent with prior studies (e.g., Chwilla and Kolk, 2005; Khalkhali et al., 2012) word recognition of targets following the thematic primes was due to a global integration process as suggested by the correlations with BEAGLE cosines (and to a marginal extent with LSA) at the longer 500 and 800 ms SOAs. Finally, as in Jones and Mewhort (2007), we also found that BEAGLE cosines predicted taxonomic priming. Given that the BEAGLE model's distributed representation extends beyond shared features to also include abstracted representations such as co-exemplars and category labels, it is perhaps not too surprising that BEAGLE cosines should predict taxonomic priming. In turn, this underlying predictor suggests that taxonomic relations are retrospectively activated following target presentation as posited by the semantic matching model (Neely, 1991) and post-lexical integration model (e.g., De Groot, 1984, 1985). Feature similarity may also facilitate this process (McRae and Boisvert, 1998; Thompson-Schill et al., 1998). Though not significant, there was a trend in the predicted inverse direction between feature similarity and word recognition for the taxonomic targets across all three SOAs. The lack of significance is most likely due to the range restriction for feature similarity among the taxonomic items (i.e., >4.00 on a seven-point scale). With greater variation in similarity among taxonomically related items such as the items used by McRae and Boisvert (1998), feature similarity would likely also be a reliable predictor of target word recognition.

### Implications for behavioral and corpus studies

Association strength poses an additional variable that should be examined further. In our current research, we focused on only weakly associated integrative, thematic, and taxonomic pairs, because the associative boost found in prior studies with taxonomic pairs (Moss et al., 1995; for review see Lucas, 2000; Hutchison, 2003; Jones and Estes, 2012) and integrative pairs (Jones, in preparation) may mask the more subtle differences on other measures among these relations. However, one worthy avenue of pursuit would be to compare and contrast the relative impact association strength has on priming effects within each relation across a variety of LDT paradigms favoring perceptual simulation, spreading activation, or expectancy generation. Such an investigation would require a large set of items having equivalent means and variability of association strength across the three relations. The selection of items from large scale studies provide the advantage of larger item sets with a greater variability in the measures of interest, whereas smaller created item sets having the same targets have the advantage of more experimental control.

### Conflict of interest statement

The authors declare that the research was conducted in the absence of any commercial or financial relationships that could be construed as a potential conflict of interest.

## Appendix A

**Table:** **Study 1 Stimuli**.

**Integrative Pairs**			
joint account	pest control	picnic lunch	executive secretary
head ache	nut cracker	copy machine	customer service
carbon atomv	computer disc	flea market	hair shampoo
snack attack	swan dive	jello mold	loan shark
data base	fashion fad	designer name	traffic sign
waste basket	football field	identification number	deposit slip
asteroid belt	mailbox flag	quart oil	laundry soap
cork board	antique furniture	cave opening	train station
gravy boat	net gain	dust pan	stick stone
ankle bracelet	herb garden	cuff pants	cross street
medicine cabinet	career goal	research paper	raspberry tart
birthday candle	camp ground	ice pick	property tax
health care	pool hall	nail polish	vodka tonic
sand castle	department head	will power	mountain top
brain cell	pledge honor	frog prince	bath towel
breakfast cereal	gray hound	bullet proof	personality trait
graduation ceremony	mint jelly	plum pudding	moral values
lawn chair	ski jump	equal rights	stair way
pipe cleaner	shop keeper	cardinal rule	cob web
comedy club	shoe lace	potato salad	trade wind
ethics code	sign language	tuna sandwich	
press conference	brick layer	skill saw	
mass confusion	shore line	split second	
Thematic Pairs			
swamp alligator	couple date	bush leaves	sphinx pyramid
jungle animal	caravan desert	storm lightning	alligator river
medieval armor	physical doctor	vault lock	burglar robbery
fort army	evacuate earthquake	roach motel	tar roof
trombone band	siren emergency	rocks mountain	shovel sand
suds bath	headband exercise	physician needle	darkness scared
pillow blanket	home family	bat night	sailor sea
wedding bride	rooster farm	patient nurse	playground slide
herd buffalo	nun father	sponge ocean	kettle stove
desert cactus	sailing fishing	nurse patient	grief tears
cupboard cans	path forest	elephant peanut	cop ticket
blocks children	steak fries	fountain penny	bank vault
balloon clown	softball girls	sentence period	blood vein
sugar coffee	pasture grass	aircraft pilot	chef waiter
stadium concert	onion hamburger	buffalo plain	marriage wedding
crane construction	wheat hay	luggage plane	fireplace winter
cape coral	ape jungle	ace poker	giraffe zoo
accuse court	stove kitchen	algae pool	
haystack cow	otter lake	chapel priest	
spank cry	grass lawn	limousine prom	
Taxonomic Pairs			
pronoun adjective	stag doe	branch leaf	broccoli spinach
lead aluminum	pound dollar	cougar lion	raccoon squirrel
wrist ankle	zebra donkey	shrimp lobster	veal steak
frustration anxiety	mongoose duck	yard mile	canal stream
handbag backpack	eye ear	compound mixture	lagoon swamp
jazz ballet	notebook folder	brother mother	bus taxi
baseball basketball	insight foresight	glands neck	rain thunder
cello bass	spatula fork	cousin nephew	muffin toast
bedroom bathroom	mink fox	nerve neuron	squash tomato
sofa bed	moss fungus	garlic onion	lip tongue
bathroom bedroom	colonel general	refrigerator oven	plaque trophy
champagne beer	brandy gin	drawing painting	soup vegetables
abdomen body	envy greed	carnival party	ceiling wall
broom brush	forehead hair	pear peach	south west
spinach cabbage	thigh hip	comet planet	rye wheat
turnip carrot	antler horn	metal plastic	cotton wool
ceramics crafts	cattle horse	bowl plate	editor writer
chalk crayon	earthquake hurricane	gallon quart	meter yard
cone cup	sweater jacket	mortgage rent	week year
son dad	swimmer jogger	pub restaurant	
moose deer	relish ketchup	plate saucer	
shelf desk	rope ladder	tenor soprano	

## Appendix B

### Instructions for rating tasks

#### Thematic relatedness

For each word pair (e.g., CREAM COFFEE; BLACKBOARD CHALK), you will be asked to rate the extent to which the concepts are linked together in a common scenario, event, or function. For example, CREAM is often added to COFFEE, and CHALK is used to write on a BLACKBOARD. Thus, these items are thematically connected to each other. Please note that thematically connected items may not necessarily share similar features. For example, BLACKBOARD and CHALK are different shapes and sizes and are made out of different materials. For each pair, rate the extent to which the pair is connected to some common theme (such as a classroom theme in the above example) on a scale from 1 (not thematically connected) to 7 (highly thematically connected). Please use the full range of the scale (1, 2, 3, 4, 5, 6, or 7) in indicating your responses.

#### Integrative ratings (sentence context)

You will judge the sensibility of a word pair (shown in ALL CAPS) within its sentence (e.g., George picked up the CEREAL BOWL or George picked up the PLATE BOWL). Please indicate your sensibility judgment on a scale from 1 (not at all sensible) to 7 (completely sensible). Please use the full range of the scale (1, 2, 3, 4, 5, 6, or 7) in indicating your responses.

#### Category relatedness ratings

You will rate the extent to which the two words (e.g., NECKLACE BRACELET, DUCK GOOSE) belong to the same specific category (e.g., BIRDS rather than the more general category ANIMALS). Please rate each word pair from 1 (not at all category co-members) to 7 (definitely members of the same specific category), and be sure to use the full range of the scale (enter 1, 2, 3, 4, 5, 6, or 7). For example, NECKLACE and BRACELET are both types of jewelry and thus would likely be given a high rating. In contrast, SILVER and BRACELET belong to different categories (SILVER is a type of metal, whereas BRACELET is a type of jewelry) and thus should be given a lower rating. Some items (HORSE CHICKEN) will belong to the same general category (HORSES and CHICKENS are both animals) but should also be given a lower rating as horses are a type of mammal and chickens are a type of bird.

#### Familiarity ratings

In the following experiment you will read a series of 195 word pairs (e.g., CREAM COFFEE; BLACKBOARD CHALK). For each word pair, please rate how unfamiliar or familiar the word pair is. For example, a word pair such as FRUIT BASKET might sound more familiar to you than the word pair DONKEY HILL. The scale ranges from 1 (unfamiliar) to 7 (familiar). Please use the full range of the scale (1, 2, 3, 4, 5, 6, or 7) in indicating your responses.

#### Feature similarity ratings

You will rate the similarity of the two words (e.g., DOTS STRIPES) on a scale from 1 (not at all similar) to 7 (very similar). Please use the full range of the scale (1, 2, 3, 4, 5, 6, or 7) in indicating your responses.

Two words are similar if they look alike or belong to the same category. For example, DOTS and STRIPES are similar (both are types of patterns or designs). However, SHIRT and STRIPES would not be similar. Even though stripes are often found on shirts, a shirt is a type of CLOTHING. Furthermore, whereas ZEBRA is associated with STRIPES, these two words are also not very similar, because they belong to different categories (i.e., animal and pattern categories).

## Appendix C

**Table:** **Stimuli**.

**Target**	**Prime-type**		
	**Integrative**	**Thematic**	**Taxonomic**
bacon	turkey	eggs	pork
book	instruction	editor	article
breakfast	hotel	bacon	lunch
cake	fruit	party	muffin
candy	chocolate	halloween	gum
carrot	garden	salad	beet
cat	alley	pet	lion
clock	antique	hour	watch
closet	broom	hanger	cabinet
coat	wool	lab	cape
cow	barn	dairy	pig
crown	jewel	queen	hat
doctor	animal	prescription	dentist
fries	carnival	steak	chips
guitar	strings	concert	drums
heart	donor	blood	liver
horse	trail	wagon	cow
hotdog	beef	mustard	sausage
jar	glass	jelly	bottle
lamp	street	bulb	flashlight
needle	steel	thimble	thorn
ocean	coral	shark	lake
organ	pipe	church	accordion
oyster	sea	pearl	scallop
panda	jungle	bamboo	grizzly
pants	linen	hem	suit
pencil	art	notebook	crayon
prison	inmate	guard	dungeon
rain	summer	hurricane	sleet
river	forest	boat	lake
robe	cotton	bath	cloak
saxophone	brass	jazz	clarinet
ship	battle	harbor	yacht
shirt	silk	tie	jacket
siren	ambulance	emergency	alarm
skill	job	expert	technique
skirt	suede	girl	shorts
smoke	signal	pollution	smog
soap	dishwasher	shower	shampoo
soccer	field	kick	volleyball
soup	tomato	bowl	chili
speech	history	campaign	lecture
spoon	silver	tea	knife
sport	contact	coach	tennis
